# Hydro-dynamic Solute Transport under Two-Phase Flow Conditions

**DOI:** 10.1038/s41598-017-06748-1

**Published:** 2017-07-26

**Authors:** Nikolaos K. Karadimitriou, Vahid Joekar-Niasar, Omar Godinez Brizuela

**Affiliations:** School of Chemical Engineering and Analytical Science, Faculty of Engineering and Physical Science, University of Manchester, M13 9PL Manchester, United Kingdom

## Abstract

There are abundant examples of natural, engineering and industrial applications, in which “solute transport” and “mixing” in porous media occur under multiphase flow conditions. Current state-of-the-art understanding and modelling of such processes are established based on flawed and non-representative models. Moreover, there is no direct experimental result to show the true hydrodynamics of transport and mixing under multiphase flow conditions while the saturation topology is being kept constant for a number of flow rates. With the use of a custom-made microscope, and under well-controlled flow boundary conditions, we visualized directly the transport of a tracer in a Reservoir-on-Chip (RoC) micromodel filled with two immiscible fluids. This study provides novel insights into the saturation-dependency of transport and mixing in porous media. To our knowledge, this is the first reported pore-scale experiment in which the saturation topology, relative permeability, and tortuosity were kept constant and transport was studied under different dynamic conditions in a wide range of saturation. The critical role of two-phase hydrodynamic properties on non-Fickian transport and saturation-dependency of dispersion are discussed, which highlight the major flaws in parametrization of existing models.

## Introduction

Transport in porous materials is important for many industrial applications and natural processes such as transport of chemicals in enhanced oil recovery, fertilizers in agricultural soils, salt water intrusion into fresh water coastal aquifers, groundwater contaminations, and deliverance of pharmaceutical products to live tissues^1–7^. It is clear that in many of these applications more than one fluid phase is present in porous media﻿. This means that in these cases the theories and parameterization of transport under single - fluid phase flow are not valid and applicable. Under single-f-luid phase conditions, hydro-dynamic dispersion (*D*) is empirically proposed to be a power law function of average pore-scale velocity (u), defined as *D* = *D*_*m*_ + *u* ^*n*^ ^8^. In this relation, *D*_*m*_ is the molecular diffusion coefficient, and α is the diffusivity coefficient. Obviously α and *n* are both empirically determined, where the latter one is usually proposed to be equal to unity. This relation has been simply generalized for transport under two fluid phase flow by assuming the saturation-dependency of the proposed parameters *D*(*S*) = *D*_*m*_ + (*S*)*u* ^*n*(*S*)^ ^9^. Obviously this formulation does not provide any new insights into the fundamental two-fluid phase transport processes, and can even lead to an over-estimation of dispersion coefficient for a given saturation^10–13^.

Despite the fundamental importance and serious applied implications of understanding two fluid phase transport in porous media, it has received far less attention compared to the single - fluid phase transport. From a macro-scale perspective, several researchers investigated transport phenomena under unsaturated (two fluid phase) conditions^14–20^. However, lessons learnt from these studies are based on indirect estimations of hydro-dynamic transport properties and fitting “empirically-proposed models” to experimental data.

In contrary to the column-scale experiments^17, 20, 21^, high-resolution real-time (optical and X-ray) imaging has lately allowed us to quantify directly the pore-scale mixing and transport in porous media^22–24^ even under two fluid phase conditions^25, 26^. From the hydro-dynamic point of view, the existence of two fluid phases in a porous medium will create some hydrodynamically stagnant zones in each fluid with very weak (close to zero) velocity fields, which will make the local transport regime to be diffusive. This is in contrast to the remaining area occupied by the same fluid where transport is advective-dispersive. Based on this dual characteristic of the pore network, some mechanistic models such as dead-end pore model^27^, and the mobile-immobile model (MIM)^10, 28^ have been developed. These two models employ similar assumptions as they account for mobile and immobile regions at a given saturation, and a diffusive mass transfer between these regions^9, 29^.

Major drawbacks in MIM have been pointed out by ref. 25 *Karadimitriou et al*.^25^ who directly quantified the stagnant zones and showed that the diffusive mass exchange rate between the mobile and the immobile region in the MIM model is time and flow-rate dependent. Also, although the MIM model could fit the experimental data very well, there was no consistency between the fitted parameters and parameters estimated directly from the experiments. This study is a follow-up study of *Karadimitriou et al*.^25^ in which for the first time using direct image analysis, the impact of flow rate on transport properties under the same saturation topology is studied.

The objective here is to employ a direct experimental method (without inverse modelling) to estimate (a) the impact of the Péclet number on the stagnant saturation, (b) examine the relation between the stagnant saturation and total saturation, (c) to provide physically-based explanation for the huge data scattering in stagnant saturation reported the literature. In this paper we aim to present direct estimation of stagnant saturation versus total saturation to investigate whether under well-defined conditions, a meaningful relation (which is missing in the literature) between them exists. It is important to note that we have established a novel experimental procedure such that for a given saturation without any change in saturation topology, we have managed to examine the transport properties at different ink injection rates (reproducing different Péclet numbers). Then, we directly identified the dispersion coefficients of the flowing saturation versus the total saturation and its relation with saturation. To our knowledge, this unique finding provides the first direct assessment of transport under two fluid phase flow conditions. As the continuation of the work of *Karadimitriou et al*.^25^, we conducted 3 sets of dynamic two-phase flow experiments to establish a saturation topology in an elongated micro-model, which comprised 4 independent representative elementary volumes (REVs). While we kept the saturation topology undisturbed, we managed to perform injection of water-based ink in a two-phase (water-Floruinert) micro-model at 4 different rates (each injection rate producing a different Peclet number). In this way, we produced 48 REV-based data sets.

In this paper, after a short review of the experimental methods and data analysis methodology, novel results focused on hydro-dynamic transport under two fluid phase conditions based on direct analysis are presented and discussed.

## Materials and Methods

### Experimental Setup

The experimental setup, image processing and data analysis used in this work are almost similar to the ones used in the work of *Karadimitriou et al*.^25^. We used a similar Poly-Di-Methyl-Siloxane (PDMS) micro-model, Fluorinert FC-43 as the wetting phase, water as the non-wetting phase, and water-based ink as the dispersed phase, respectively. The micro-model was 4 REV long and each REV was visualized and recorded by a dedicated mono-chrome 5 Megapixel camera synchronically. For more information on the micro-model and the visualization setup, please refer to the ref. 30 and SI.

The micro-model was initially fully saturated with Fluorinert. Then, water was introduced into the flow network at a given flow rate (0.2 to 0.5 ml/h) to establish a steady-state saturation. After breakthrough of water in the micromodels, the saturation and its topology would not change unless a very high rate was imposed. To avoid any internal remobilization after reaching the steady-state saturation pattern with non-dyed water, dyed-water was introduced at a low flow rate of 0.1 ml/h. Meanwhile we visualized the saturation field to make sure it did not change. The water-based ink and fluorinert are immiscible and the water-based ink got dispersed only in the area occupied by water. Meanwhile, the transport of the ink into the flow network was visualized and recorded. Then, flow was inversed and water was reintroduced into the flow network at the same flow rate until all the ink had been washed away. This process of introduction and removal of ink was carefully performed such that the saturation topology does not change. This allowed us to maintain exact fluids topology to perform another ink injection experiment at a higher rate. The process was repeated for a number of flow rates for the introduction and removal of ink, namely 0.2, 0.6, and 1 ml/h. In total, 3 two-phase flow experiments were performed (resulting in 12 different saturations per REV) and ink injection and removal were performed at 4 different rates.

### Image Processing and Data Analysis

The images acquired from the four individual cameras, each focusing on a different REV, were used to extract information on phase saturation, resident concertation, and the identification of flowing and stagnant regions.

#### Saturation

In order to identify saturation of each phase in the flow network, a “*saturation mask*” of the steady-state distribution of the fluid phases in the network was prepared. In this way it was possible to isolate the area occupied exclusively from water, as this was the area where ink would be dispersed. By assigning different grayscale intensity values to the pixels belonging to the water-filled area, and Fluorinert-filled area, these two phases were distinguished in the saturation mask.

#### Resident Concentration

Resident concentration was estimated in the same way as reported in *Karadimitriou et al*.^25^. A nonlinear calibration between grayscale intensity and local concentration was used to convert the grayscale intensity in the images to real concentration values. All images were processed in order to have a uniform illumination, and they were cross-correlated to the saturation mask. Materials (e.g. PDMS, Fluorinert, water and ink) and the optical setup were identical to the ones employed in *K* *aradimitriou et al*.^25^, where more detailed information on the methodology can be found.

#### Flowing and Stagnant Regions

To identify the flowing and stagnant regions in the water-filled part of the flow network, for the fastest ink injection rate (1 ml/h) we identified the time when the temporal gradient of concentration averaged over the REV (*C*′(*t*)) would take its maximum value, referred to as *t*_max_. Based on the 1-D Ogata-Bank analytical solution for concentration versus time, 2*t*_max_ is the characteristic time when the Fickian transport in the flowing part of the network has saturated the flowing network. Due to the very small characteristic transport time in the flowing part of the network, compared to the very large characteristic transport time in the stagnant zone, the mass transfer from the flowing part of the network to the stagnant one is negligible for this ink injection rate. Then, we refer to the image taken at time equal to 2*t*_max_ (with respect to the ink entrance time). In this image, those pixels carrying concentration belong to the flowing part of the network, and those pixels which do not carry ink belong to the stagnant zone.

#### Calculation of Dispersion Coefficient and Average Pore Velocity

We continuously injected ink into the water-Fluorinert filled network of the micro-model. Ink dispersed only in the area occupied by water, as stated before, while the cameras were monitoring the dispersion throughout the whole extent of the flow network. Based on the acquired images we averaged the ink concentration over each REV to obtain average concentration versus time data. As explained above, in the area occupied by water there were two hydro-dynamically different regions (i.e. flowing and stagnant) with two distinct characteristic transport time scales. Therefore, the extracted data showed the non-Fickian transport in longer time scales controlled by diffusion in the stagnant regions. The early-time data were fitted by the one-dimensional advection-dispersion analytical solution^31^ as follows:

1 $$C(x,t)=\frac{1}{2}{C}_{0}[erfc(\frac{x-ut}{2\sqrt{Dt}})+{e}^{\frac{xu}{D}}erfc(\frac{x+ut}{2\sqrt{Dt}})]$$

Note that the average resident concentration in the flow network under two fluid phase conditions does not reach the inlet concentration C_0._ Thus, for fitting of the results C_0_ will be the maximum average resident concentration measured under a given condition. Obviously, in a fully-saturated medium the maximum resident concentration should correspond to the concentration at the boundary of the pore structure. This is a straight-forward consequence of the fact that the pore network is fully percolating by design.

### Data availability

The data supporting the conclusions can be obtained in the tables provided in the supplementary document.

## Results and Discussion

**Figure 1:**
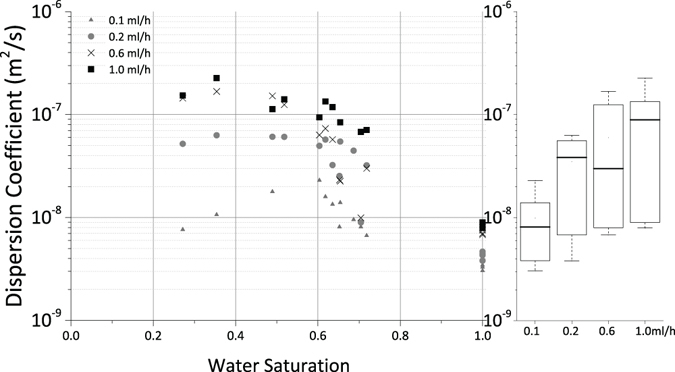
Left: Dispersion coefficient versus saturation. Right: variation of dispersion coefficient versus flow rate.

In Table S1 (in SI) the results regarding saturation, pore scale velocity, dispersion coefficient, and estimated maximum concentration in the part of the network occupied by water, are shown. These results correspond to every individual REV for all transport experiments. The dispersion coefficient, pore scale velocity, and maximum concentration were estimated by fitting equation (1) to the experimental data. The accuracy of the fitting results was estimated based on the normalized mean square error (NMSE). In all cases, it was larger than 0.97. NMSE was defined as $NMSE=1-{\Vert ({X}_{i}-{\hat{X}}_{i})/({X}_{i}-\bar{{X}_{i}})\Vert }^{2}$ in MATLAB documentation, where ||·||^2^ indicates the 2-norm of a vector. *X*_*i*_, $\overline{{X}_{i}}$ and ${\hat{X}}_{i}$ are the experimental data, average of the experimental data, and fitting data, respectively. The dispersion coefficients (log scale) versus saturation for different ink rates for the total area occupied by water and the advective part of it are shown in Fig. 1.

One can see a clear non-monotonic trend between dispersion coefficient and saturation at a flow rate of 0.1 ml//h, which maximizes at a saturation of 0.6. At higher flow rates this non-monotonicity diminishes.

For the lowest injection rate, the advective transport in the flowing part of the water-filled part of the network is comparable to the diffusive flow between the flowing and the stagnant part of it, and the variation of the dispersion coefficient with saturation is small. As the pore velocities increase for increasing ink injection rates, dispersion (rather than diffusion) gradually becomes the dominant transport mechanism. This can be interpreted as the reason for which the variation of the dispersion coefficient increases with respect to saturation with increase of ink injection rate (i.e. Péclet number defined as the ratio of advective to diffusive flux), as shown in Fig. 1 (right). Since *et al*. flow rates the saturation and tortuosity cases are identical, the impact of the dynamics of two-phase flow on the variation of the dispersion coefficient is not relevant. However it is visible that at small saturation values (0.27 and 0.34) the range of variation of the dispersion coefficient is much more pronounced compared to the fully-saturated case. This suggests that there is no unique Péclet number-dispersion coefficient relation for a given porous medium, and this relation is saturation dependent.

**Figure 2:**
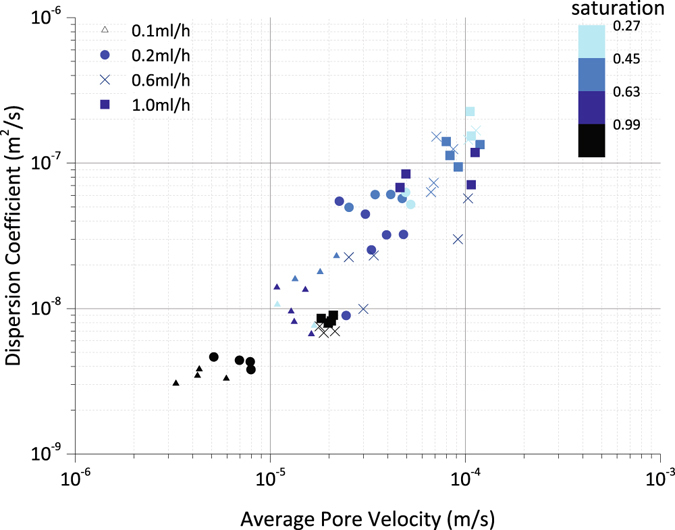
Dispersion coefficient plotted against average pore velocity, for the four different injection rates. The data points are attained by fitting Equation (1) to the average resident concentration versus time data. Colors coding shows the saturation class of each data point. Black color shows the fully-saturated cases and cyan color shows the saturation of 0.27. The other two blue colors show the intermittent ranges of saturation.

In order to explore the saturation-dependency of dispersion coefficient versus pore-scale velocity relation, we plotted the results obtained by fitting the Equation (1) to the experimental data in Fig. 2. As expected, under two-phase flow conditions the pore-scale velocity increases in comparison to a fully saturated network. In addition to this, for an increasing pore-scale velocity, the corresponding dispersion coefficient also increases. However, looking at the data points and their variation, it is clear that the scatter of the data is highly saturation-dependent, as shown by color coding of 4 saturation classes in Fig. 2.

The applicability of the generalized power law relation between pore scale velocity and dispersion relation, D(S) = D_m_ + (S)u^n(S)^, is questionable. Out fitting results shown in Figure S2 show that the assumption that both *α* and *n* terms are only functions of saturation is not well supported. There is an erratic relation between the parameters and saturation that indicates saturation alone is not definitely enough to relation saturation and dispersion coefficient and backbone saturation tortuosity may need to be included.

**Figure 3:**
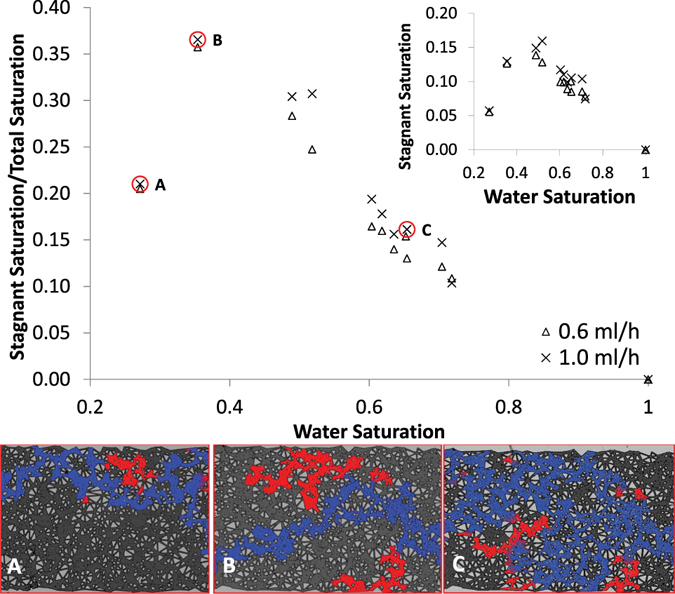
Ratio of stagnant saturation over total saturation versus the total saturation for the two fastest ink injection rates of 0.6 ml/h (Δ) and 1.0 ml/h (×). Inset: stagnant saturation versus the total saturation. Stagnant (red) and flowing (blue) water-filled networks of the micro-model at three different saturations (**A**,**B**,**C**) have been shown in the color figures.

As discussed earlier the stagnant regions created by the two fluid phase configuration control the non-monotonicity in dispersion coefficient-saturation relation and the saturation-dependency of the dispersion coefficient-pore velocity relation. Thus, we need to directly estimate the stagnant saturation created at different water saturation states. Following the methodology explained earlier, we estimated the stagnant saturation in the two fastest flow rates as shown in Fig. 3 (tabulated data can be found in Table S2).

Figure 3 shows a clear parabolic behavior with identical trends between stagnant saturation and total saturation for ink injection rates of 0.6 and 1 ml/h. Since the hydro-dynamically stagnant and flowing water part of the network in a laminar regime is independent of the flow rate, the relation between stagnant saturation and total saturation should be flow-independent. This has been verified in Fig. 3 as for the two injection rates, the trend is flow-independent. This clear unique trend provides new valuable insights into the two-phase transport processes and helps improving predictive modelling. Till to date the relation between stagnant saturation and total saturation was re-constructed using fitting of the models (e.g. MIM) to the experimental data^13^. Thus, from a physical point-of-view the obtained results from the existing models could be potentially not representative and even incorrect^25^. Due to the significant discrepancy of literature data, no conclusion could be drawn about this trend.

Again, we notice that at saturation close to 0.35 the higher stagnant fraction is resulted. The saturation at which stagnant saturation fraction maximizes corresponds to the maximum dispersion coefficient obtained in Fig. 2. This non-monotonicity is not trivial as hydro-dynamic dispersion in the flowing network should increase monotonically with increase of pore velocity (due to decrease of water saturation) under a constant injection rate boundary condition. However the stagnant water network which is diffusion-controlled has a non-monotonic trend with saturation. This implies that the non-monotonic stagnant saturation controls the non-monotonicity in the effective dispersion of the two-phase medium. The functionality among the dispersion coefficients of the whole water network and flowing water network, total water saturation and stagnant water saturation require more detailed and extensive study of transport under two-phase condition, which will be the focus of future studies.

## Implications for industrial applications

The results of this study have serious implications for industrial applications among which enhanced oil recovery is a major one. In various enhanced oil recovery methods, such as surfactant flooding or low salinity water flooding, transport of chemicals through a two- or three-phase system is the central process. However, modelling of transport in this multiphase system is still in its infancy. Our results show that in order to improve the predictive modelling capabilities, a new theoretical modeling framework is needed to integrate the non-monotonic saturation-dependency of dispersion coefficient and stagnant saturation integrated into two-phase dynamic simulators. Obviously, the existence of stagnant regions will prevent the transport of chemicals to the target zones (even in rather homogenous reservoirs) with the time scale of water flooding, which will impede the performance of the technology. Moreover, in applications such as low salinity water flooding^32, 33^, the resident water(referred to as formation brine) has a very different composition compared to the chemically- designed injected water. Thus, the mixing between the formation brine as the injected water, which is a slow process, will deteriorate the quality of the injected water and the oil recovery performance. Based on the findings of this paper, stagnant saturation is highly variable with saturation which means the mixing time-scale between stagnant and flowing water will be saturation dependent and oil recovery performance will alter by saturation.This adds a new dimension to the complexity of the "low salinity water flooding" process. On top of the previously established concepts related to to the impact of the petrophysical properties at large scale and the critical role of surface force interactions of crude oil-brine-rock at sub micron scale, the stagnant regions will play also an important role in the performance of the low salinity water flooding at an intermediate scale.

## Conclusions

This study presents a micro-model experimental research to investigate the transport mechanism under two-phase conditions in porous media. For a given saturation, saturation topology, tortuosity, and effective permeability were kept constant for all Péclet numbers in order to eliminate their effect on the distribution of the experimental data. While keeping tortuosity constant, it focuses on the existence of any relation between stagnant saturation and total saturation and the correlation between the stagnant saturation and dispersion coefficient.

Based on the results of the micro-model experiments, the following key conclusions can be withdrawn:

- The dispersion coefficient shows a clear non-monotonic trend with saturation, especially at smaller Peclet numbers, which maximizes at intermediate saturations. The dispersion coefficient under two-phase conditions can be one order of magnitude larger than that of single-phase flow.
- At different saturation values, ranging from 0.27 to 1.0, we have been able to establish a steady-state saturation topology and inject ink tracer at four different rates. That allowed us to investigate the impact of Péclet number on dispersion coefficient keeping the tortuosity and effective permeability constant.
- Keeping the tortuosity and effective permeability constant at a given saturation, flow does not impact the relation between stagnant saturation and total saturation, which is in agreement with theoretical understandings. For laminar linear flow, the stagnant saturation should be independent of flow rate and it should be only a function of saturation topology. That highlights that a theoretical framework needs to be developed such that for a given saturation topology the stagnant and flowing volume fractions (i.e. immobile and mobile saturations) are independent from flow hydro-dynamics.
- Our results show that the coefficients involved in the estimation of dispersion coefficient as a power law of pore scale velocity are not functions of saturation only. Further studies need to be done in order to properly establish a relation between dispersion coefficients and pore scale velocity.
- The results imply a relation between the fraction of stagnant saturation (stagnant saturation divided by total saturation) and total dispersion coefficient. The saturation, at which the dispersion coefficient maximizes, coincides with the maximum stagnant saturation fraction.

## Electronic supplementary material

Supplemdentary Information

Supplementary Tables
