# Supplementary material for: Hydro-dynamic Solute Transport under Two-Phase Flow Conditions
Source: Sci Rep. 2017 Jul 26;7:6624. doi: 10.1038/s41598-017-06748-1 (PMC5529577; doi:10.1038/s41598-017-06748-1)
Supplement: Supplementary file 1 — Supplemdentary Information [file 41598_2017_6748_MOESM1_ESM.pdf]

Supplementary Information

Hydro-dynamic Solute Transport under Two-Phase Flow Conditions

Nikolaos K. Karadimitriou, Vahid Joekar-Niasar, Omar Godinez Brizuela

School of Chemical Engineering and Analytical Science, Faculty of Engineering and Physical Science, University of Manchester, M13 9PL, Manchester, United Kingdom

Corresponding author: Vahid Joekar-Niasar (vahid.niasar@manchester.ac.uk)

Table S1. Flow rate, saturation, dispersion coefficient, pore velocity and maximum concentration with NMSE with respect to REV.

Flow rate (ml/h)	Saturation	REV	Dispersion (m2/s)	Pore velocity (m/s)	Cmax	NMSE
**0.1**	0.69	**1**	**9.6E-09**	**3.2E-07**	**0.92**	**0.979**
**0.1**	0.65	**2**	**1.4E-08**	**2.7E-07**	**0.96**	**0.988**
**0.1**	0.70	**3**	**8.1E-09**	**3.3E-07**	**0.82**	**0.985**
**0.1**	0.65	**4**	**8.1E-09**	**3.3E-07**	**0.82**	**0.988**
**0.1**	0.62	**1**	**1.6E-08**	**3.4E-07**	**0.91**	**0.981**
**0.1**	0.64	**2**	**1.4E-08**	**3.8E-07**	**0.85**	**0.985**
**0.1**	0.72	**3**	**6.7E-09**	**4.1E-07**	**0.93**	**0.991**
**0.1**	0.60	**4**	**2.3E-08**	**5.5E-07**	**0.91**	**0.999**
**0.1**	0.35	**2**	**1.1E-08**	**2.7E-07**	**0.65**	**0.997**
**0.1**	0.27	**3**	**7.6E-09**	**4.2E-07**	**0.88**	**0.992**
**0.1**	0.49	**4**	**1.8E-08**	**4.5E-07**	**0.75**	**0.992**
**0.1**	1.00	**1**	**3.1E-09**	**8.2E-08**	**1.00**	**0.999**
**0.1**	1.00	**2**	**3.3E-09**	**1.5E-07**	**1.00**	**0.998**
**0.1**	1.00	**3**	**3.5E-09**	**1.1E-07**	**1.00**	**0.999**
**0.1**	1.00	**4**	**3.8E-09**	**1.1E-07**	**1.00**	**0.998**
**0.2**	0.69	**1**	**4.5E-08**	**7.7E-07**	**0.96**	**0.972**
**0.2**	0.65	**2**	**5.5E-08**	**5.7E-07**	**0.99**	**0.98**
**0.2**	0.70	**3**	**9.0E-09**	**6.1E-07**	**0.81**	**0.98**
**0.2**	0.65	**4**	**2.5E-08**	**8.3E-07**	**0.86**	**0.98**
**0.2**	0.62	**1**	**5.7E-08**	**1.2E-06**	**0.85**	**0.98**
**0.2**	0.64	**2**	**3.2E-08**	**1.2E-06**	**0.87**	**0.987**
**0.2**	0.72	**3**	**3.2E-08**	**9.9E-07**	**0.88**	**0.985**
**0.2**	0.60	**4**	**5.0E-08**	**6.3E-07**	**0.93**	**0.986**
**0.2**	0.52	**1**	**6.1E-08**	**8.6E-07**	**0.79**	**0.98**
**0.2**	0.35	**2**	**6.3E-08**	**1.2E-06**	**0.61**	**0.982**
**0.2**	0.27	**3**	**5.2E-08**	**1.3E-06**	**0.95**	**0.986**
**0.2**	0.49	**4**	**6.1E-08**	**1.0E-06**	**0.74**	**0.99**
**0.2**	1.00	**1**	**4.4E-09**	**1.7E-07**	**1.00**	**0.999**
**0.2**	1.00	**2**	**3.8E-09**	**2.0E-07**	**1.00**	**0.999**
**0.2**	1.00	**3**	**4.7E-09**	**1.3E-07**	**1.00**	**0.999**
**0.2**	1.00	**4**	**4.3E-09**	**2.0E-07**	**1.00**	**0.999**
**0.6**	0.65	**2**	**2.3E-08**	**6.3E-07**	**0.98**	**0.99**
**0.6**	0.70	**3**	**9.9E-09**	**7.5E-07**	**0.78**	**0.997**
**0.6**	0.65	**4**	**2.3E-08**	**8.5E-07**	**0.84**	**0.99**
**0.6**	0.62	**1**	**7.3E-08**	**1.7E-06**	**0.88**	**0.982**
**0.6**	0.64	**2**	**5.7E-08**	**2.6E-06**	**0.89**	**0.984**
**0.6**	0.72	**3**	**3.0E-08**	**2.3E-06**	**0.86**	**0.989**
**0.6**	0.60	**4**	**6.3E-08**	**1.7E-06**	**0.88**	**0.99**
**0.6**	0.52	**1**	**1.3E-07**	**2.2E-06**	**0.77**	**0.994**
**0.6**	0.35	**2**	**1.7E-07**	**2.8E-06**	**0.60**	**0.991**

**0.6**	0.27	**3**	**1.4E-07**	**2.6E-06**	**0.94**	**0.99**
**0.6**	0.49	**4**	**1.5E-07**	**1.8E-06**	**0.73**	**0.985**
**0.6**	1.00	**1**	**7.0E-09**	**5.4E-07**	**1.00**	**0.998**
**0.6**	1.00	**2**	**6.8E-09**	**4.7E-07**	**1.00**	**0.997**
**0.6**	1.00	**3**	**8.0E-09**	**4.9E-07**	**1.00**	**0.999**
**0.6**	1.00	**4**	**7.6E-09**	**4.5E-07**	**1.00**	**0.99**
**1**	0.65	**2**	**8.4E-08**	**1.2E-06**	**0.68**	**0.977**
**1**	0.70	**3**	**6.8E-08**	**1.2E-06**	**0.54**	**0.979**
**1**	0.62	**1**	**1.3E-07**	**3.0E-06**	**0.89**	**0.986**
**1**	0.64	**2**	**1.2E-07**	**2.8E-06**	**0.89**	**0.988**
**1**	0.72	**3**	**7.1E-08**	**2.7E-06**	**0.84**	**0.977**
**1**	0.60	**4**	**9.4E-08**	**2.3E-06**	**0.86**	**0.982**
**1**	0.52	**1**	**1.4E-07**	**2.0E-06**	**0.67**	**0.99**
**1**	0.35	**2**	**2.3E-07**	**2.6E-06**	**0.55**	**0.99**
**1**	0.27	**3**	**1.5E-07**	**2.7E-06**	**0.85**	**0.989**
**1**	0.49	**4**	**1.1E-07**	**2.1E-06**	**0.58**	**0.989**
**1**	1.00	**1**	**8.0E-09**	**5.0E-07**	**1.00**	**0.997**
**1**	1.00	**2**	**8.2E-09**	**5.1E-07**	**1.00**	**0.998**
**1**	1.00	**3**	**9.0E-09**	**5.3E-07**	**1.00**	**0.997**
**1**	**1.00**	**4**	**8.6E-09**	**4.6E-07**	**1.00**	**0.999**

Table S2. Flow rate, saturation, and immobile saturation over total saturation with respect to REV.

Flow rate (ml/h)	Saturation	REV	Immobile/total saturation
**0.1**	0.69	**1**	0.12
**0.1**	0.65	**2**	0.17
**0.1**	0.70	**3**	0.17
**0.1**	0.65	**4**	0.16
**0.1**	0.62	**1**	0.16
**0.1**	0.64	**2**	0.19
**0.1**	0.72	**3**	0.06
**0.1**	0.60	**4**	0.14
**0.1**	0.35	**2**	0.34
**0.1**	0.27	**3**	0.21
**0.1**	0.49	**4**	0.26
**0.1**	1.00	**1**	0.00
**0.1**	1.00	**2**	0.00
**0.1**	1.00	**3**	0.00
**0.1**	1.00	**4**	0.00
**0.2**	0.69	**1**	0.14
**0.2**	0.65	**2**	0.15
**0.2**	0.70	**3**	0.13
**0.2**	0.65	**4**	0.16
**0.2**	0.62	**1**	0.16
**0.2**	0.64	**2**	0.16
**0.2**	0.72	**3**	0.10
**0.2**	0.60	**4**	0.19
**0.2**	0.52	**1**	0.24
**0.2**	0.35	**2**	0.35
**0.2**	0.27	**3**	0.20
**0.2**	0.49	**4**	0.28
**0.2**	1.00	**1**	0.00
**0.2**	1.00	**2**	0.00
**0.2**	1.00	**3**	0.00
**0.2**	1.00	**4**	0.00
**0.6**	0.65	**2**	0.13
**0.6**	0.70	**3**	0.12
**0.6**	0.65	**4**	0.15
**0.6**	0.62	**1**	0.16
**0.6**	0.64	**2**	0.14
**0.6**	0.72	**3**	0.11
**0.6**	0.60	**4**	0.17
**0.6**	0.52	**1**	0.25
**0.6**	0.35	**2**	0.36

**0.6**	0.27	**3**	0.21
**0.6**	0.49	**4**	0.28
**0.6**	1.00	**1**	0.00
**0.6**	1.00	**2**	0.00
**0.6**	1.00	**3**	0.00
**0.6**	1.00	**4**	0.00
**1**	0.65	**2**	0.16
**1**	0.70	**3**	0.15
**1**	0.62	**1**	0.18
**1**	0.64	**2**	0.16
**1**	0.72	**3**	0.10
**1**	0.60	**4**	0.19
**1**	0.52	**1**	0.31
**1**	0.35	**2**	0.37
**1**	0.27	**3**	0.21
**1**	0.49	**4**	0.30
**1**	1.00	**1**	0.00
**1**	1.00	**2**	0.00
**1**	1.00	**3**	0.00
**1**	1.00	**4**	0.00

Micro-model

The micro-model used in this work was a Poly-Di-Methyl-Siloxane **(**PDMS) one. The dimensions of the flow network were 5 × 30 mm^2 with a constant depth of 38 micrometres, and a mean pore size of 70 micrometres. The flow network topology was based on Delaunay triangulation, as it is considered to be a good approximation of natural porous media (1). The flow network had 5,000 pore bodies and nearly 15,000 pore throats. The size of the REV, which has been reported in (2), was determined to be 5 × 5 mm^2, casting the flow network more than four REVs long. In order for the micro-model to have a uniform and stable wettability, before it initial use it was slianized in order to become hydrophobic, as described in (3).

Visualization Setup

The major components of the visualization setup are shown in Figure S1. Numbers are used so as to facilitate their identification.

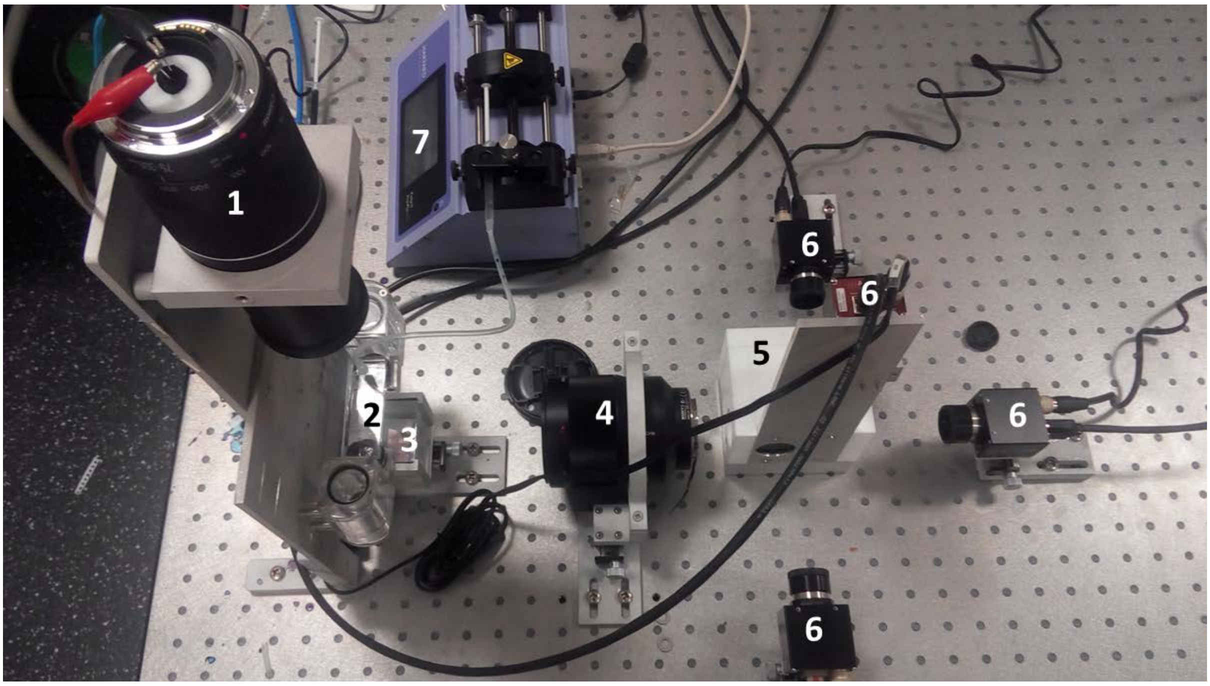

Figure S1: The major components of the visualization setup are shown.

The major components of the optical setup - labelled by numbers in Figure S1- are explained here: a collimated LED light source mounted with an objective lens F 3.2/105 mm (1), a prism (Edmund Optics) with dimensions of 50 mm × 50 mm (3), a SONY Sonnar F1.8/135 mm magnifying lens (4), a box containing three beam splitters (Edmund Optics) with dimensions of 35 mm × 35 mm (5), and four 5-Megapixel cameras (Prosilica GC-2450)(6). More information on the visualization setup can be found in (2).

Power law relation between dispersion coefficient and pore velocity under unsaturated conditions

To examine the empirical relation of $D(S) = D_m + \alpha(S)u^{n(S)}$, the equation was fitted to the simulation results. As Figure S2 shows α and n are strongly changing with saturation. However, in the range of saturation of 0.6 to 0.7 there is clear drop in the values, which cannot be from physical point-of-view. As expected, saturation value is not enough to capture the relation between D and u.

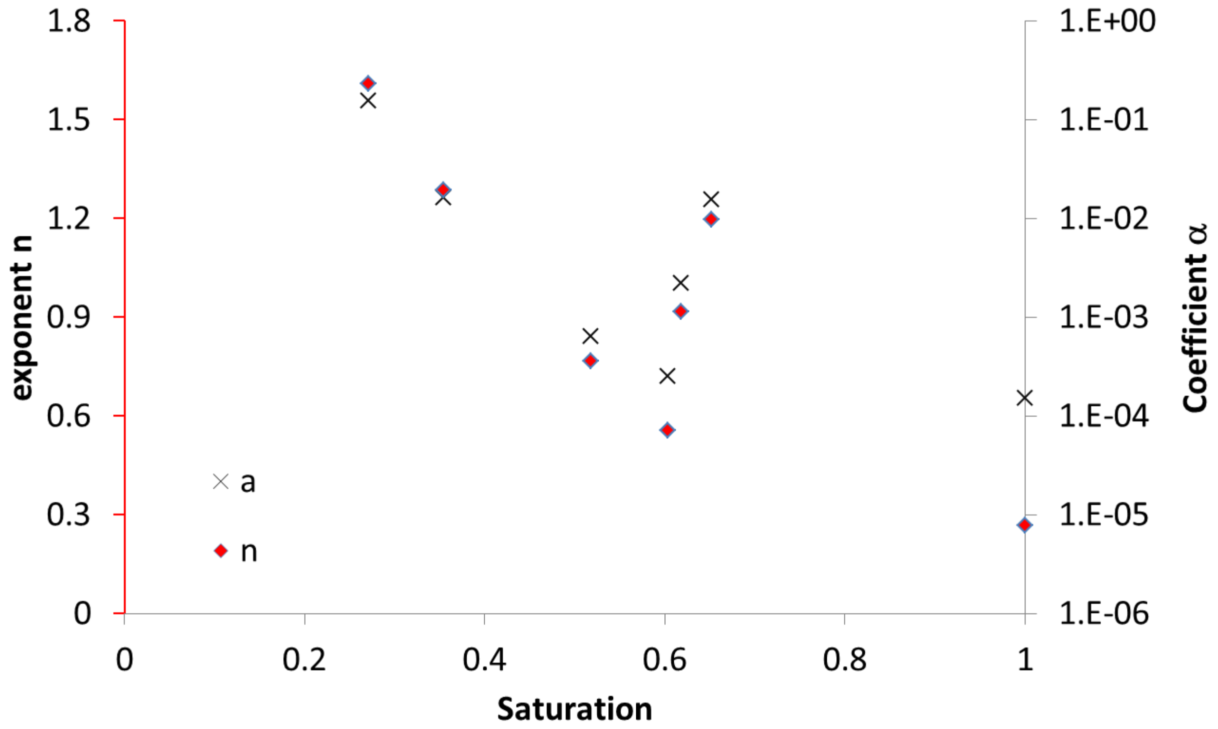

Figure S2: Coefficient α (in log scale) and exponent term n (in linear scale) are plotted versus saturation as proposed in $D(S) = D_m + \alpha(S)u^{n(S)}$. Only those values have been presented which have R^2 larger than 0.8.

References

1. Helba AA, Sahimi M, Scriven LE, Davis HT (1992) Percolation theory of two-phase relative permeability. *SPE Reservoir Engineering* 7(01):123–132.
2. Karadimitriou NK, Joekar-Niasar V, Hassanizadeh SM, Kleingeld PJ, Pyrak-Nolte LJ (2012) A novel deep reactive ion etched (DRIE) glass micro-model for two-phase flow experiments. *Lab on a Chip* 12(18):3413–3418.
3. Karadimitriou NK, et al. (2013) On the fabrication of PDMS micromodels by rapid prototyping, and their use in two-phase flow studies. Water Resources Research 49(4):2056–2067.
